# Perceived key change phenomena of MDMA-assisted psychotherapy for the treatment of severe PTSD: an interpretative phenomenological analysis of clinical integration sessions

**DOI:** 10.3389/fpsyt.2023.957824

**Published:** 2023-07-07

**Authors:** Macha Godes, Jasper Lucas, Eric Vermetten

**Affiliations:** ^1^Institute of Psychology, Social Science Department, University of Amsterdam, Amsterdam, Netherlands; ^2^Institute of Psychology, Social Science Department, Leiden University, Leiden, Netherlands; ^3^Department of Psychiatry, Leiden University Medical Center, Leiden, Netherlands

**Keywords:** PTSD, interpretative phenomenological analyses, psychotherapy, MDMA, MDMA-assisted psychotherapy, qualitative research, psychedelics

## Abstract

Post-traumatic stress disorder (PTSD) is a prevalent psychiatric condition that significantly impacts daily functioning in patients but lacks adequate treatment options. 3,4-methylenedioxymethamphetamine (MDMA) as an adjunct to psychotherapy for the treatment of PTSD has been studied increasingly for the last two decades and has shown promising results through quantitative data. However, few qualitative studies have been conducted to investigate patients’ experiences who participate in these trials. This study intends to complement and clarify the quantitative findings resulting from a Phase-II clinical trial for assessing the safety and efficacy of MDMA-assisted psychotherapy for PTSD by using a qualitative approach based on available material of 4 recorded and transcripted integrative sessions per participant. An Interpretative Phenomenological Analysis (IPA) was conducted for 7 participants who met criteria for severe PTSD to develop a deeper understanding of the treatment and its efficacy. Analysis results provided real-life statements from participants that reflect perceived mechanisms of change and showed to what extent their proposed working mechanisms integrate into daily life.

## Introduction

Post-traumatic stress disorder (PTSD) is an impairing anxiety disorder marked by re-experiencing phenomena, affect dysregulation, hypervigilance, and, most importantly, fear and avoidance associated with recalling traumatic memories (1). Studies on existing treatments have demonstrated that only 50–60% of individuals no longer meet PTSD criteria in most successful trials, leaving behind nearly half of the studied population with chronic PTSD that gain little to no benefit from current treatments (2–4).

For the last two decades, 3,4-methylenedioxymethamphetamine (MDMA) -assisted psychotherapy (MDMA-AP) has regained interest as a promising therapy for severe therapy-resistant PTSD (5–8). Multiple of these trials have primarily focused on collecting quantitative data necessary for mapping the safety and efficacy of MDMA-AP (5, 9–11). While quantitative measures successfully address the intensity and frequency of PTSD symptoms, they fail to provide a deeper phenomenological understanding of the treatment process. Consequently, they cannot fully capture the potential benefits that may arise during the treatment. A qualitative research approach may yield a more comprehensive view of the issue being studied and enables exploration of respondents’ inner experience (12, 13). To date, relatively little attention has been devoted to qualitative studies on MDMA-AP as a clinical practice to examine how patients perceive the therapy to work and describe key phenomena related to symptom improvement and behavioral change. Previous work includes Barone and colleagues’ paper from 2019, which explored long-term follow-up sessions of MDMA-AP for veterans, police officers, and firefighters (14).

This study is the first to explore how participants experience change and processing of trauma after undergoing MDMA-AP by assessment of recorded clinical integration sessions. It is also explored how participants integrate the therapeutic elements of the therapy into their daily life during the course of the treatment.

## Methods

For this study, MAPS provided video material of therapy sessions conducted for the phase 2 open-label clinical trial for assessing the safety and efficacy of MDMA-AP for severe PTSD (15). For a detailed report of quantitative outcomes of this phase 2 trial, see Multidisciplinary Association for Psychedelic Studies (16).

**Figure 1:**
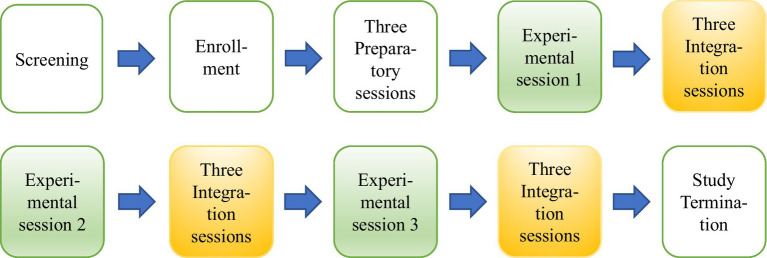
Study design of MDMA assisted psychotherapy. Experimental sessions shown in green. Integration sessions shown in yellow.

**Figure 2:**
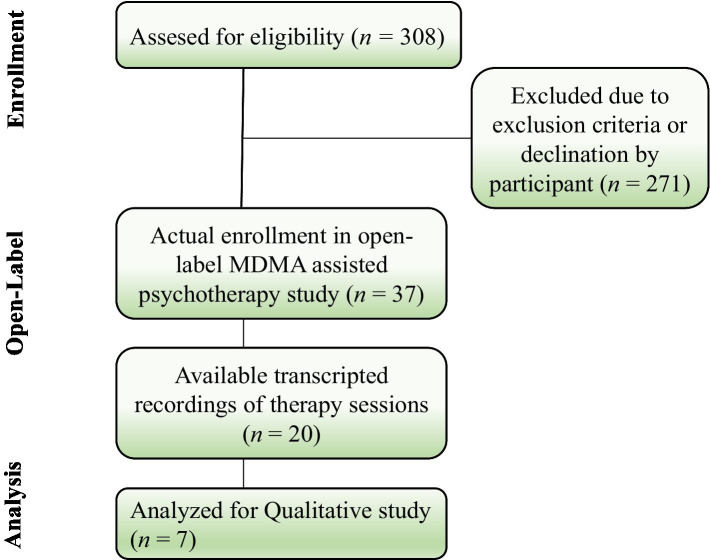
Diagram denoting flow of participants throuhout the study.

Each participant underwent a recorded treatment consisting of 15 sessions divided over 12 weeks. The treatment protocol embodied three different types of sessions (Figure 1). Three experimental MDMA sessions took place in which either 80 mg or 120 mg is orally administered with an optional supplemental dose of 40 mg or 60 mg. In these 6–8-h long sessions, addressing PTSD symptoms and processing the trauma is an essential part. After each MDMA session, three integration meetings took place. See Figures 1, 2 for the treatment design and procedure. More information on the treatment procedure and protocol is found in the ‘Manual for MDMA-AP for the treatment of post-traumatic stress disorder (15).

**Figure 3:**
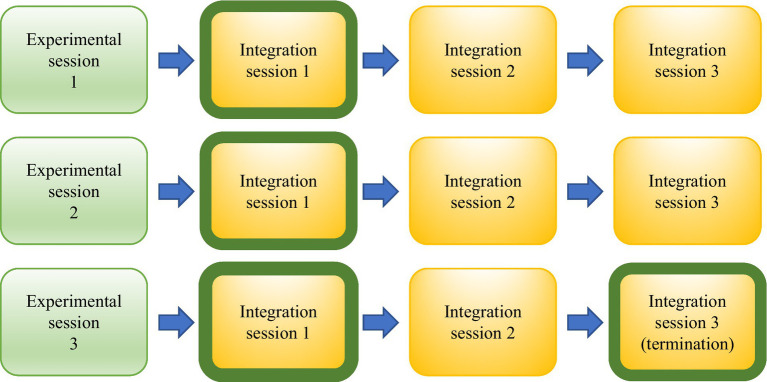
Selected integration sessions per participant for qualitative analysis (shown with green border).

To explore and understand the content, quality, and meaning of participants’ descriptions after undergoing MDMA-AP, transcribed video recordings of the first therapeutic integration sessions right after the experimental sessions and the last of all sessions were analyzed using a qualitative approach (Figure 3). The integration sessions included in this analysis were part of a complete clinical treatment, and no structured qualitative interviews were conducted apart from the therapeutic intervention.

### Coding and analysis

For analysis, Interpretative Phenomenological Analysis (IPA) (17) was used. IPA aims to provide a “detailed examination of personal lived experience, the meaning of experience to participants and how participants make sense of that experience” (18) and, therefore, make sense of the changes they go through in response to the treatment. In essence, the inquirer undertakes several steps to reduce the data and attempts to capture the phenomena that all participants hold in common. IPA typically holds an idiographic component which allows the researcher to look for the meaning behind an individual’s experience by interpreting the data through a psychological lens (19). Also, IPA is one of the primary methodologies used in similar clinical trials focusing on MDMA and alternate compounds (14, 20, 21).

After the recorded treatment sessions were transcribed, they underwent thematic analysis by the three-member research team. The coding team (MG and JL) constructed an initial coding frame, which was then reviewed and discussed by the auditor (EV). The encoding process was based on the step-by-step protocol of conducting IPA as proposed by Pietkiewicz and Smith (19).

### Participants

Initially, 308 participants were assessed for eligibility for the open-label pilot study of MDMA-AP for severe PTSD. After screening, 37 participants actually enrolled in the study. From those 37 participants, there were video recordings available from 20 participants. Five participant videos were excluded due to unusable video and sound quality. Given practical matters such as time limitation and taking Robinsons’ guidelines for choosing a sample size for a qualitative IPA study into consideration, a subset of 7 participants was chosen by applying stratified sampling (22, 23). In doing so, four women and three men were randomly chosen after dividing the full sample into males and females. An overview of demographic characteristics, initial CAPS-5 scores and CAPS-5 scores after the treatment are shown in Tables 1, 2. A diagram presenting the flow of participants from the phase 2 clinical trial to the current study is shown in Figure 2.

**Table 1:** Demographic characteristics for 7 participants in the qualitative analysis.

Fictive name	Gender	Age	Ethnicity	Cause of PTSD	CAPS-5 baseline	CAPS-5 termination
Sophie	Female	25	White	Developmental trauma/multiple trauma	43	23
Isa	Female	43	White	Developmental trauma/multiple trauma	47	5
Rose	Female	23	White	Developmental trauma/multiple trauma	55	14
Jacky	Female	40	White	Developmental trauma/multiple trauma	38	5
Nick	Male	50	White	Veteran/combat exposure	41	13
Rico	Male	32	Asian	Veteran/combat exposure	53	13
Harry	Male	41	White	Developmental trauma	47	10

**Table 2:** Demographic characteristics for initially enrolled participants and subset.

	Total (*N* = 37)	Subset (*n* = 7)
Gender, *n* (%)		
Male	15 (40.5)	4 (57.15)
Female	22 (59.5)	3 (42.85)
Age, M (SD)	35.6 (10.8)	36.3 (9.19)
Min, Max	18.6, 62.3	23, 50
Ethnicity, *n* (%)		
American Indian	1 (2.7)	0
or Alaskan native		
Asian	6 (16.2)	1 (14.3)
Black	1 (2.7)	0
White	27 (73.0)	6 (85.7)
Multiple	2 (5.4)	0
Type of trauma, *n* (%)		
Developmental trauma	29 (78.4)	5 (71.4)
Veteran	5 (15.2)	2 (28.6)
Combat exposure	3 (9.1)	2 (28.6)
Multiple trauma	27 (81.8)	4 (57.1)
CAPS-5, baseline (*n* = 37), M (SD)	45.4 (7.18)	46.28 (5.72)
Change in CAPS-5, baseline to termination (*n* = 36), M (SD)	−29.89 (13.45)	−34 (15.71)

One participant withdrew from the trial before to the onset of the second MDMA-AP session due to a mild increase of nightmares. This individual was the only one who did not complete the primary endpoint evaluation.

## Results

In total, 2,240 min of recorded video material from seven participants’ integration sessions were coded and analyzed. All seven participants reported experiencing a range of benefits during the course and at the end of the treatment. The presented coding schema (Table 3) entails the data’s main categories and themes and served as a guiding framework. All quotes illustrating the themes of the coding scheme are found in the supplementary materials. For data to be valid and reportable, it was decided that each theme had to be reported by at least four participants.

**Table 3:** Coding scheme denoting all categories and themes for 7 participants.

Coding scheme		
Categories	Themes	Participants per theme
Tolerance of conflict	Staying with what ‘is’	7
	Decreased reactivity	6
Processing trauma	Insight, reflection, linking	7
	Mental clarity	4
	Recovery of traumatic memories	5
	Disentangling trauma from self	5
	Reuniting lost affects and parts	4
Positive emotions	Self-acceptance	7
	Joy, happiness, gratitude	7
	Hope and Empowerment	7
	Relaxation, calmness, peace	7
Interpersonal	Comfort	5
	Gratitude, empathy, compassion	4
Connection	Union, wider perspective	4
	Inner healing intelligence	6
	Accessibility to emotions	5
	Mind–body connection	4

### Tolerance of conflict

#### Staying with what ‘is’

This theme refers to the participants’ ability to stay with, observe, and acknowledge a feeling before responding or reacting in the way one usually would do. This may be accompanied by a sense of curiosity concerning the meaning and source of the emotion. Rather than attempting to suppress the feeling through distraction or other methods, participants now take the time to notice the feeling, sit with it for a while, and trust and allow it to dissipate. They recognize the transient and subjective nature of emotional states, resulting in greater flexibility and adaptability. Sophie, for example, strikingly describes the ability to not only tolerate painful memories but actually continue the healing process in those moments:

“I feel like even with our session on Saturday, even though it was difficult, and there was a lot of pain. I let myself experience that pain again. I don’t feel like any progress had been robbed from it. I feel like probably more progress have been made from it. And that’s like the same thing with, if any unwanted memories come up, now it’s an interesting kind of re-evaluating of an unwanted memory, because I know in this process I’m trying to heal and understand and remember the totality of what has happened.”

#### Decreased reactivity

In this theme, participants report experiencing diminished reactivity and engagement with aversive thoughts, memories, and daily encounters. Also, experiences relating to increased tolerance towards others, especially towards elements that would previously evoke an aversive reaction and negatively affect the participant, were reported. Some participants described these diminished feelings as ‘spontaneous,’ while others experienced them as a result of insights or emotional processing during the MDMA sessions.

### Processing trauma

#### Insight, reflecting

This theme comprehends the degree of reflective thinking or insights gained regarding psychological processes. This reflective thinking may be related to relational or internal patterns, cultural norms, or specific insights about the traumatic encounter and PTSD symptoms. For example, participants’ descriptions included pivotal insights about the trauma origin and their behavior in response to the traumatic content. Participants also showed an increased capacity to put formerly ineffable complexities into words. Nick, for example, describes a greater understanding of what the trauma ‘took from him’.

“I was thinking last night that a lot did connect for me and starts to make sense for me that I’ve never really been able to put together before. (…) I think that part softened for me, knowing what it meant to me. Mentally knowing that that damage was done to me. That softened, I feel like now that I know what it took from me, I know where to begin to rebuild, and just finding that out almost gave me back a little bit of data that I felt they stole from me. Because now I have a starting point. I think all of this is going to build on my self-confidence and love for myself and love for others and appreciation for life.”

#### Mental clarity

This theme depicts participants’ experiences of a clearer understanding of complex concepts and life situations. Participants showed an increased ability to explore feelings and traumatic memories with a degree of focus and coherence that is usually out of their reach, often leading to new insights. This theme can further be characterized by participants’ experiences of a mental ‘fog’ or ‘cloud’ disappearing, enabling them to think and reason more clearly.

#### Recovery of traumatic memories

This theme refers to participants’ ability to retrieve previously inaccessible memories. Participants reported spontaneous recovery of traumatic memories that they had largely or completely forgotten about. This recovery was often accompanied by a critical insight into their trauma history and how it shaped their current behavior.

#### Disentangling trauma from self

For this theme, participants described a sense of separation from their trauma and the behavioral patterns it caused them to adopt. They somehow managed to recognize how these patterns may have served them in the past while also recognizing how they cause dysfunction in their current lives. This theme includes quotes from participants reflecting on their trauma as a separate construct from the larger “being” they identify with - almost like an uninvited friend they had to deal with but for whom it is now time to leave. The following quote from Isa showcases her realization that she had internalized her trauma and need not to do so any longer:

“I really felt like it wasn’t part of me or attached to me anymore. And I feel like it made me realize the totality of everything that’s happened. I’ve made it mine and my fault or something. I’ve made it part of me. You know, and the ugliness and the craziness of it all. I made it mine. Like I’m that monster, evil I’m that, you know. But it wasn’t me. It was just something that happened I guess I don’t know. It’s not me, it just happened to me, it’s like some violent thing happens to somebody.”

#### Reuniting lost parts

This theme describes participants’ experiences of remembering or relating to an aspect of the self that had previously been lost, locked-up or split off, rejected, suppressed, controlled, or amputated by the participant, often as a result of the traumatic experience.

“After that first session there were, in my mind’s eye, two individuals. That were the 15-year-old Harry and there was the present-day Harry. They were distinct bodies but at the end of yesterday, they went together and that was really the connection that I was looking for. I felt so good. There was the unification integration.”

### Positive emotions

#### Self-acceptance

This theme refers to participants’ feelings of appreciation, acceptance, compassion, and empathy for themselves. This includes feeling more secure when rejected by others and reduced feelings of guilt and shame. Participants described that these feelings of guilt and shame often arose from the belief that they had played a responsible role in the traumatic event(s). Participants’ quotes selected for this theme also include descriptions of forgiveness and care towards oneself.

#### Joy, happiness, gratitude

An essential aspect of the increase in positive emotions that participants describe is increased joy and gratitude. They report being enabled to enjoy pleasurable activities again without interference from their anxiety. Also, they report an increased ability to enjoy life or certain aspects of life again. In addition, they express greater gratitude towards their life circumstances and those they share them with. Besides the burden that the symptoms of PTSD have been in their lives, they saw now how these experiences can enrich them as a person.

#### Hope and empowerment

This theme reflects participants’ experiences of self-confidence, empowerment, and hope. They look forward to continuing the therapeutic process after the trial has finished as they experience a sense of trust, empowerment, and confidence in their ability to defend their boundaries or manage other challenging situations. Participants state feeling in control of one’s own life and experiencing the freedom to stay with a feeling or react according to one’s preference. Rico expressed his experience of this theme using a metaphor:

“I kind of had this vision of a traditional brick and mortar stacking, this wall, finally sealing off. And what I saw, it’s like more softened and I can just push these bricks out. I can predict the bricks to dismantle the wall at the pace that I need to do it, the wall is still there, but I can take down the pieces of it at my own pace. And I can see what’s beyond it again.”

#### Relaxation, calmness, peace

This theme illustrates participants’ enhanced ability to relax and calm down, where they usually would have been alert and tense. Participants also described a sense of relief in their body, particularly in areas of the body that had previously been in pain, tension, or tightness.

### Interpersonal

#### Comfort

This theme refers to an increased sense of comfort in accepting help and love from others. Participants report feeling more secure in seeing to it that their emotional needs are met, in addition to increased levels of intimacy. Participants describe a diminishing of obstacles or defenses that previously impeded social closeness. Also, participants noted how trusting and opening up positively add to the deepening of social connections with others, which Rose describes as follows:

“I was stressing out, and he was trying to give me advice. And never before could I confidently say that somebody could give me advice, and I would sit there and try to listen to what they’re saying. Usually I’m just like, no, you’re wrong when you don’t understand, but like I was actively, believing him and wanting his advice and I was able to see it from that perspective. You know, but like before I was so stuck that I couldn’t listen to what they were saying.”

#### Gratitude, compassion, empathy

Participants’ examples of appreciation, gratitude, forgiveness, and compassion for others are illustrated in this theme. Also included are participants’ expressions of interest in volunteering or peer counseling. Finally, participants felt that they wanted to share what they have gained throughout the therapy and, in this way, serve others, PTSD sufferers in particular.

### Connection

#### Union, wider perspective

This theme refers to an increased sense of union that participants report with the “universe,” their surroundings, and others. Also, a sense of ‘awe’ at the vastness of the world and universe is described. These feelings seemed to provide participants with a broader perspective on their problems. This can involve recognizing the common human plight that they share with others and feeling a sense of belonging in the world.

#### Inner healing intelligence

Here, participants’ report sensing the presence of an “inner-healing intelligence” or “guide” within themselves, which, once recognized and connected with, can aid in healing and recovery. Participants reported feeling stronger connected to this semi-autonomous part of their psyche. When connecting with this mysterious intelligence, it seems to open up a path to recovery and healing. In the following quote, Sophie emphasizes the ‘magical’ and ‘esoteric’ elements of MDMA-AP.

“When you say, ‘the inner healer’ I think that the medicine (MDMA) allows something within you to find that awaiting ability to express it, whatever that something is. So, there’s definitely a almost esoteric sense to it, for lack of a better term, there’s some kind of magic to that, of you getting in that spot and be like, “hey, remember this?” And it’s not always a good thing. But it’s there. It’s being brought up for a reason. And that to me is like, whoa. Yeah, truly It really is. It’s like a moment of like, Oh, yeah, there’s this memory. That’s there for a reason. What is it bringing me? What is it teaching me and why is it still prevalent? What does it mean?”

#### Accessibility to emotions, in contact with feelings

This theme illustrates participants’ experiences of acknowledging or coping with previously rejected or inaccessible feelings and self-states. These feelings and self-states may reflect vulnerability, tenderness, love, happiness, carefree, playful, and a broader and deeper level of feeling in general.

#### Mind–body connection

Participants linked to this theme referred to specific bodily sensations and how they relate to their trauma. This may include connecting a particular bodily state with an emotional state or reconnecting to one’s bodily state at the time of a traumatic event. Also, an overall sense of connecting more to the body was repeatedly stated. Experiencing this connection between mind and body allowed for a greater understanding of their trauma, facilitating new ways of adapting to and dealing with its consequences.

## Discussion

This study is the first to explore how participants experience change and relief of symptoms after undergoing MDMA-AP by investigating recorded clinical integration sessions. Results include statements of how patients perceive, experience, explore and process challenging emotions previously avoided or blocked. It explored how individuals with severe PTSD experience change and relief of symptoms after undergoing MDMA-AP and how the underlying therapeutic elements were integrated into their daily lives. We attempted to portray the participants’ integration experience through the lens of psychological growth and therapeutic action and capture which mechanisms of action might have played a role. We will now briefly expand on our views concerning the interrelatedness of the different themes.

**Figure 4:**
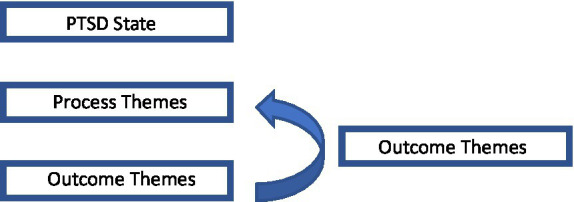
Schematic representation denoting the interrelatedness of identified themes.

At the risk of attempting to organize such complex, subjective material into an overly rigid theoretical framework with heterogeneous and overlapping constructs, we would nonetheless like to categorize the found categories and their subthemes in ‘process’, ‘outcome’ and ‘growth themes’. The ‘process’ themes, which include the categories ‘Tolerance of conflict’ and ‘Processing trauma’, refer to psychological processes that lead to ‘outcome’ themes, which include the categories ‘Positive emotions’ and ‘Interpersonal’. For example, in our view, the ‘process’ subtheme ‘Staying with what is’ may actually facilitate the outcome subtheme ‘Comfort’. We make this distinction in part because of the deep sense that MDMA-AP is by no means a magic bullet: MDMA opens patients up to engage in psychological processes that lead to beneficial outcomes and thereby requires considerable effort from the patients. However, a short-coming of this distinction is that the participants in our study also almost invariably describe the sense that their therapeutic work will continue for a long time following their participation in the trial. To recognize this aspect of our findings, we would like to formulate the third and final category of growth themes, which includes the category ‘Connection’. ‘Growth’ themes refer not to processes that occur as a result of MDMA administration, but to abilities that participants take with them from the experience and continue to apply in daily life. These growth themes seem to allow participants to once again engage in processes similar to those they experienced during their MDMA sessions. For example, participants reported increased ability to connect to their feelings, as described by the subtheme ‘Accessibility to emotions’. This might allow participants to once again engage in the process of ‘staying with what is’ in daily life after the treatment has ended, thereby further facilitating outcome themes. See Figure 4 for a schematic representation of our views on the possible interrelatedness of our identified themes.

The main findings of this research offered several insights that well complement and expand upon the quantitative outcomes of the study it is based on. The Primary Category ‘Positive Emotions’ in particular shows how the benefits of MDMA-AP may well go beyond symptom reduction, as noted by Barone and colleagues as well (14). Our findings also offer support for existing theoretical models that aimed to explain how MDMA’s physiological and psychological effects might mediate the treatment of PTSD. The themes ‘mental clarity’ and ‘staying with what is’, for example, correspond well to speculation about the role of down regulation of limbic structures and upregulation of prefrontal areas in the brain in MDMA-AP’s therapeutic effects (24).

Furthermore, confronting the painful event and allowing for memory updating and modifying the fear response plays an essential role in most trauma-focused therapies (25, 26). Previous research by Mithoefer et al., (27) found that MDMA-AP provides a desirable state of altered perception in which the psychological root cause of PTSD symptoms may be addressed, and fear extinction and memory reconsolidation of threatening memories may be facilitated. Supporting this theory, the current study found how certain elements of this state may have remained an accessible resource throughout the treatment, even after the physiological effects of MDMA had drawn off. During and after constructing the coding scheme, it became apparent that the participants’ increased ability to ‘stay with what is’ and tolerate whatever came up ran like a thread throughout the relevant data. Participants often stated that feelings of joy, compassion, decreased defenses and improved introspection and communication resulted from an increased ability to endure otherwise painful states. Their increased ability to relax and stay present in the moment seemed to create space for participants to direct their energy toward other important aspects of life that would previously be inaccessible.

Moreover, this study found how participants may recover from ‘moral injury’. Moral injury refers to the violation of moral beliefs that create a deep sense of internal estrangement and conflict (23). The guilt and shame associated with moral injury often form an obstacle in participating in therapies (23). The participants’ statements showed an increase in acceptance, self-forgiveness, and self-empathy, which are key in addressing moral injury and the feelings of guilt and shame that tie to it.

Further, the results portray how MDMA-AP impacts daily functioning regardless of the relative change in CAPS-5 scores. The FDA’s decision to approve MAPS to proceed forward with phase 3 clinical studies was mainly based on the sustained improvement in CAPS-5 scores seen in phase 2 trials. A change in CAPS-5 scores, however, can only present the frequency and intensity of symptoms and cannot account for other factors that affect daily functioning and quality of life as a result of having PTSD. In our sample, all 7 participants showed clinically significant decreases in PTSD symptoms at trial termination compared to baseline CAPS-5 scores, with an average change in CAPS-5 total scores of 34 (*SD* = 8), ranging from a difference of 20 (Sophie) to 42 (Isa; Figure 1). Although the difference in both scores is relatively high, both participants state that they experienced a range of additional outcomes apart from features addressed by the CAPS-5, including increased self-acceptance and an enhanced capacity to reflect on themselves. Outcomes like these not only contribute by discerning specific features of MDMA-AP that play an essential role in symptom improvement, but also help in better customize future studies and inform and improve therapy effectiveness.

### Limitations

This study presents several limitations that warrant consideration. Firstly, participants reported challenges in articulating their post-therapy experiences, as they were still processing the internal experiential changes. Consequently, the best available spoken language of participants was employed to develop an appropriate coding scheme. Participants may benefit from additional time and distance to gain a more comprehensive understanding of their experiences. Secondly, a larger team conducting the qualitative analysis would have been ideal. The smaller team size may have led to subjective overinterpretation, influenced in part by enthusiasm regarding the method. A more extensive team could have helped mitigate this risk. Thirdly, the varied MDMA dosage per individual might have resulted in substantial differences in response and subsequent experiences. The concentration of MDMA in the body is a critical determinant of its effects (28). Every participant received 80–120 mg initially and had an option to receive an additional dose of 60 mg after 3 h into the session. The research team was unable to access the individual dose range, which constitutes a major limitation to this study.

The findings of this study emphasize the importance of qualitative research in studying MDMA-AP by complementing and clarifying its quantitative outcomes. The themes reflected by participants aid in a better understanding of the known theoretical frameworks that sought to explain this therapy’s working mechanism. Future research could use this study’s findings in designing better fitting protocols in order to expand psychological mechanisms of action and maximize therapeutic benefit.

## Data availability statement

Raw data can only be requested and granted by obtaining a confidentiality agreement with MAPS, as the data contains highly sensitive and private data of the participants. Further inquires can be directed to the corresponding author.

## Ethics statement

The studies involving human participants were reviewed and approved by Western Copernicus Group Independent IRB (Research Triangle, NC), University of California San Francisco Human Resource Protection Program IRB, University of Madison Wisconsin Health Sciences IRB, Western IRB (Puyallup, WA), and University of British Columbia Providence Health Care Research Ethics Board. The patients/participants provided their written informed consent to participate in this study. Written informed consent was obtained from the individual(s) for the publication of any potentially identifiable images or data included in this article.

## Author contributions

MG wrote and coded and designed the entire material. MG designed the coding scheme together with JL. JL coded the entire material and helped designing the coding scheme. EV held an overview and gave critical feedback during the entire process. All authors contributed to the article and approved the submitted version.

## Funding

This Clinical Trial was sponsored by the Multidisciplinary Association for Psychedelic Studies (MAPS), a 501(c)(3) nonprofit organization. MAPS provided the MDMA and fully funded this study from private donations. MAPS Public Benefit Corporation (MAPS PBC), wholly owned by MAPS, was the trial organizer. Also, MAPS provided the funding for publishing this article.

## Conflict of interest

The authors declare that the research was conducted in the absence of any commercial or financial relationships that could be construed as a potential conflict of interest.

## Publisher’s note

All claims expressed in this article are solely those of the authors and do not necessarily represent those of their affiliated organizations, or those of the publisher, the editors and the reviewers. Any product that may be evaluated in this article, or claim that may be made by its manufacturer, is not guaranteed or endorsed by the publisher.
