# Supplementary material for: Perceived key change phenomena of MDMA-assisted psychotherapy for the treatment of severe PTSD: an interpretative phenomenological analysis of clinical integration sessions
Source: Front Psychiatry. 2023 Jul 7;14:957824. doi: 10.3389/fpsyt.2023.957824 (PMC10361411; doi:10.3389/fpsyt.2023.957824)
Supplement: Supplementary file 1 [file Data_Sheet_1.docx]

**Supplementary material: Coding Scheme including quotes**

With each quote, the corresponding integration session is stated, in which ‘v’ stands for ‘video’ and the number following ‘v’ corresponds to the session in therapy from which the quote was drawn.

**Primary category 1: Tolerance of conflict**

*Theme 1 Staying with what ‘is’*

Sophie (25y) | v13

“It really stuck out to me that like whenever a memory or a feeling of the trauma was happening again, that's when it felt like any progress I had made in the past was just being robbed of me. And now, when memories come up from him, as processes started, I don't feel like I'm being robbed anymore of any of my emotional progress. I feel like even with our session on Saturday, even though it was difficult, and there was a lot of pain, I let myself experience that pain again. (…) And that's like the same thing with, if any unwanted memories come up, now it's an interesting kind of re-evaluating of an unwanted memory, because I know in this process I’m trying to heal and understand and remember the totality of what has happened. And so even though these memories aren’t fantastic memories or may be difficult to process or bring up some emotions of pain. They don't make me feel violated like they did before.”

Jacky (40) | v15

“I became so conditioned, I haven't even thought that I shouldn't be reacting that way or I should, you know. And so, allowing myself even if it's a millisecond, even if I still have the reaction, then thinking about it and saying, why did I react that way? Maybe next time I cannot react that way, and being present to it instead of these automatic things that just happen and I don't even think about.”

Isa (43) | v9

“After the last session, I was really able to do that. Even at night. I was sleeping well, even when I had a dream that would have been a bad dream. I was able to be conscious in the dream and just be with it and say, okay, you know, so this scary thing is coming up out of the ground, but it's okay. And then just letting it go instead of being terrified in my dreams, you know? And then like, it was just amazing. In a way, it was like being in the experimental session, you know, like every night. It was just amazing.”

Nick (41) | v5

“I was proud of myself yesterday because I let myself go. There was the part of me that's like, trying to speed through the story and fight it. Then I said to myself: I'm not going to do that. There were definitely, in my brain decision points of like, that's cool. The safety part was huge that time. Like it's okay to go back there. You're okay with it for a while. And I could see like if I tried to fight that more, that it wouldn't be as beneficial.”

*Theme 2 Decreased reactivity*

Jacky (40y) | v15

“I had that startle response. Like, it's incredibly diminished, which is insane to me. Truly, I know that there's a change in my brain because my startle responses like, almost dissipated and before it was constant, like I hear any noise and I mean, and while there was always the edge and I’d I jumped at it, you know, and I have that response, it’s an automatic response. Is not a choice.”

Sophie (25) | v13

“I'm having these memories come up and being able to write them down in the timeline thing I've been working on. And then just being able to move on with my day. And where beforehand it was just like if a memory came up or the feelings came up, I was in this hole, like I just lost it. It was like somebody pulled the carpet out from underneath me and no floor being there. There's just this giant pit that I fell into, and I'd have to try and find my way out of it every time again.”

Rose (23) | v15

“And this is what I started seeing as really interesting. Because I think before I would panic, and I wouldn't really know what I was panicking about, and I think now that I'm more coming back into my body, it's not like an all the time panic. So, whenever I am triggered like that, I can at least notice it now. And I know, because you asked me before, like, what are triggers? And I'm like, I don't know, it's like, just all the time. And I think now that it's kind of like, not an all the time thing I can kind of get out of it. You know?”

Isa (43) | V15

“What is amazing is in the past, I would have just… I would have been crying. I would have been upset I would have probably drink some sort of alcohol, you know, but I was okay. It was okay. And it was just so amazing.”

**Primary category 2 Processing Trauma**

*Theme 1 Insight, Reflecting*

Nick (41y) | v5

“I was thinking last night that a lot did connect for me and starts to make sense for me that I've never really been able to put together before. (…) I think that part softened for me, knowing what it meant to me. Mentally knowing that that damage was done to me. That softened, I feel like now that I know what it took from me, I know where to begin to rebuild, and just finding that out almost gave me back a little bit of data that I felt they stole from me. Because now I have a starting point. I think all of this is going to build on my self-confidence and love for myself and love for others and appreciation for life”

Jacky (40) | v15

“You know, this kind of stuff definitely works. (…) there is no doubt that this type of therapy does something. Because I've been in therapy for years and never made that connection. And who knows, I probably would have eventually, maybe, but it just came so clear to me. Like, no, nobody's saying anything, just the thought come to me and was like, this is why you feel this way. Very insightful. And I think that that's what it is. If I had to boil the entire experience down to like one descriptive word, it would be insightful. That's what this drug does. It allows you to see maybe your past or maybe yourself or whatever it is, in a different light, and to have like a sense of empathy for yourself in those situations. And also, some kind of excavating in your brain and maybe for some people that might seem terrifying. But for me, it's like for the first time I’m not afraid of what it is in my brain. I'm not afraid of it. Like, I know that there's some f*cking horrible sh*t in there. But I'm not afraid of it anymore. I'm just like, cool. It's in there. And so, there's some great sh*t in there all together. And so it's like, I'm not scared of it anymore. And that is incredible. Like, that's like, soaring above the clouds, you know? Because before I was just like, terrified of what was in there. When you don't know and you lack self-compassion, especially. It's like speaking a different language. Because until you have that empathy, you can't really... It's impossible - see it for what it is, you know? So yeah, I’ve definitely been feeling better.”

*Theme 2 Mental clarity*

Isa (43y) | v15

“I was realizing, I felt like the rest of the world was like this land that I didn't understand like this whole other planet, like, how do I even talk to people who It's okay to be friends with, who I can trust? I didn't know any of that, but really, I did. But it was all confused by the parasite (reference to trauma) and by the routes and all you know what I mean? It was like all this stuff was confusing. You asked me about clarity. That's what I feel like I really got, you know, I really understand it now. It's like putting glasses on, you know, I finally understand it, and I can see it clear like that.”

Rose (23) | v5

“I feel like when I was talking to my roommate in the kitchen this morning, we talked for, like an hour, and I wasn't, super worried about what she was going to say or whether she lied to me. And I wasn't, thinking like, what I was saying was wrong. And that's the biggest thing. I wasn’t thinking of saying something wrong. I could just talk like there's always what I want to say. Normally it goes into this filter of a bunch of other thoughts. It hurts your head. Yeah, so my brain was really clear. That's the biggest thing. And that's what I needed. To be able to think clearer. So, it's just pretty detailed or not even detailed, I just get rid of a lot of big things about me to make me function properly and then I can go on to detail.”

Rico 32 | v13

“There was just so much complexity, you know, all these things. And, I think even the stuff with mom and dad you know, I just feel like there's a lot more there. But I'm really happy to be here. To feel that I’ve gotten to a place of forgiveness. (…) I guess I'm less confused about it now. I gained some clarity.”

*Theme 3 Recovery of traumatic memories*

Sophie (25y) | v9

“Writing down the memories triggered all of these other memories of like all these times where I was really scared that he was going to suffocate me. And it's just crazy to me like that this happened. Almost, I mean probably every single day. (...) It was just crazy to me that I just completely like... if you'd asked me a month and a half ago about it. I would have said what are you talking about? Like, I completely didn't remember that. Wow, that's big because you know, the connection I can make with the hugging… Well, it hit me. (…) Remembering this thing about my dad is good to process. Like why I react the way I react to some people. I’s slows me down. And realizing that I let myself forget or purposefully forgot these things that were happening.”

Jacky (40) | v15

“One of the most fascinating aspects of this is truly how it's like, unlocking these recesses that you didn't even realize that you had locked up. It made me think about the terms of how do you access memory in general.”

*Theme 4 Disentangling trauma from self.*

Isa (43) | V5

“I really felt like it wasn't part of me or attached to me anymore. And I feel like it made me realize the totality of everything that's happened. I've made it mine and my fault or something. I've made it part of me. You know, and the ugliness and the craziness of it all. I made it mine. Like I'm that monster, evil I'm that, you know. But it wasn't me. It was just something that happened I guess I don't know. it's not me, it just happened to me, it's like some violent thing happens to somebody. And then maybe they think that they're the person that did it. You know, but you're not. The evil and the bad stuff that that person has done feels like it's now a part of you. I guess. It's so beautiful because then you if you think, you know, with that insight, you can imagine you can't ever have a space of compassionate witness or even insight, and it just feels like it's who you are. I've never even thought about it. Like, I never even realized that that's what I was doing. I didn't consciously even know that I was doing that. If I did, I would have said, well, that's, that's crazy. You know, don't do that. (…)

“I didn't even know where the bottom is. And I feel like I finally reached it, you know? And so, it's like, I'm not no longer like having to excavate this stuff. You know, I feel like I've gotten it out. It's like cutting the rot out of a peach or something. You know what I mean? I've gotten it out. And now I do not have to worry about all of this stuff. I can just do whatever. (…) And I could spend 10 years analyzing what was in those roots. But really, it doesn't matter because it's just all gone. It doesn't even matter what or why it's just not there anymore, you know? And it's just so great you would think that I've got to analyze every single molecule and figure out why was this here?”

Sophie (25) | v13

“Now that I see it as a thing, I think it was just like hiding in the shadows. The whole time I thought it was me. That there were thoughts going into my head and it was not me. But now I realize it like it is. It's like a whole other part of me. I feel how it got there and I just saw it as a different part of me. That was the biggest thing and I said that last time. I just saw it as not me, it's this part functioning in me.”

*Theme 5 Reuniting Lost Parts*.

Harry (50y) | v13

“After that first session there were, in my mind's eye, two individuals. That were the 15-year-old Harry and there was the present-day Harry. They were distinct bodies but at the end of yesterday, they went together and that was really the connection that I was looking for. I felt so good. There was the unification integration.”

Sophie (25) | v15

“Therapist: I wonder what's that like for you to kind of think about reuniting with that little girl or feel those honest feelings.

Sophie: It's like super amazing to have that moment, especially because when I first met the little girl, it was when I couldn't get to her like, she was somewhere, you know, in the house and I couldn't find her. And then the second time when I was in the float tank, and I met with her, I had a conversation with her but she didn't say why and we definitely didn't touch. And so, to feel like I finally was able to hold her and comfort her, almost in a sense rescue her. It feels really good. It feels like somebody's finally comforted that part of me. And it's strange and bizarre that it's myself that did that but also kind of: Who else would?

Rose (23) | v15

“Like, I know I said it before, but especially last night when I was trying to go to sleep. When I kept checking, I was Me. The word felt so much closer to me. I was me, Rose. I remember what it was like when I was a kid and the essence of how I felt. Not like, what I was doing exactly but the essence of me being inside of me. Like my aura, I guess, like feeling inside of me. I just got that back. And I kind of felt like a kid in a way. Because I feel like it has been gone for so long and it was slowly leaving me.”

**Primary category 3 Positive Emotions**

*Theme 1 Self-Acceptance*

Rico (32y) | v15

“I think having that new, more generous, more kind of caring “self” came out and that has been incredibly helpful in allowing me, more of myself. That's exciting. And I feel like I'm starting to show up for people in a way that's so different and making friends in a way that I didn't think was possible, because I didn't think I was deserving of it.”

Jacky (40) | v15

“I really do think that the majority of the healing that people are going to experience in the sessions and throughout is truly a lot of self-empathy and self-understanding. Because I think that's where it comes from. I've always considered myself a very empathetic person, but I didn't have any empathy for myself, you know, and I didn't really realize that until I went in here and then I faced it. It was almost like, my subconscious was throwing that against me constantly. And trying to figure out where that was coming from. When I was little, what set that kind of algorithm in my brain to tell me, you know, you're not good enough, whatever it was. And then basically, it's almost like the drug allows you to, I don't know, understand where that is coming from, absorb it, have empathy for it, and then through that lens, be able to see the rest of the things that have happened. So, it's almost like wiping the glass clean, right? Because then you experience things completely different. If you can be that objective because it's still yourself. If you can't be truly objective, but if you can have a space between your ego and your attachments and all the crap that has bound you down for years, and whatever the experiences, then you realize that it is just an experience. It doesn't define you, it doesn't need to define you.”

Sophie (25) | v15

“I went to yoga again, and I cried. Because I was I just so proud of myself lately, I guess. I was in the yoga class and we didn't even start yet. We just sat down and she turned the lights off. And we didn't even say “Om” yet or anything like she was just talking. And I started freaking crying. Because I started picking myself up. I was like, you know what, you are trying the best that you can. And that has made me cry guys because I don't give myself credit at all.”

*Theme 2: Joy, Happiness, Gratitude.*

Rico (32) | v15

“I was just walking down my block and looking at, you know, looking up at the trees and the sun coming through the leaves and just, being around people, talking to people. It was just such a good experience in that first week. It was strange to be able to, like feel joy like that. (…) I mean, it's good to be able to recognize positive energy moving in a restorative direction rather than focusing on the negative. I was just so transfixed on how bad everything was.”

Rose (23) | v9

“I'm starting to notice the things that make me feel joyful again. I don't even really notice it as much in the moment until after. Usually I would not be okay. And that makes me feel so happy and hopeful. And that it will work. I was not able to say that about myself in a long time. That I feel hopeful for myself. (…) It makes so much sense to me now. The most amazing thing to me in this whole experience is the thing that blew my mind and I keep talking to my boyfriend about it. It goes back to the reason why I feel so optimistic now. It’s how everything fell into place exactly how it needed to fall into place without it like scaring me too much at once or not enough or like just enough. Just perfectly perfect for me. It really blows my mind because I keep saying ‘accidentally’ perfect. But it wasn't an accident. And that's why I feel so optimistic now. My brain knows how to do that. Honestly, I'm fine because I'm just like figuring it out and I don't stress out about it.”

Sophie (25) | v15

“At the end of it, I do think that I have made some amazing, amazing progress in the last five months. I can't even put words to it. I really like myself as a human being. So, it's totally cool if he doesn't. It's really cool if anybody doesn't because I have friends that really love me and really see me for who I am. And I know my mom really loves me. And she's fine with seeing me for who I am. And at the end of the day, I love me and see me for who I really am. So, it’s cool to be a part of the dance. I can't even put it into words, like the feeling of openness and bubbliness and just like wholeness that I feel in my body. I don't feel like parts of me are missing.”

*Theme 3 Hope and Empowerment*

Rico (32y) | v15

“I kind of had this vision of a traditional brick and mortar stacking, this wall, finally sealing off. And what I saw, it's like more softened and I can just push these bricks out. I can predict the bricks to dismantle the wall at the pace that I need to do it, the wall is still there, but I can take down the pieces of it at my own pace.  And I can see what's beyond it again. There’s that fear part of what's next, you know, what's my next hurdle and there’s another wall on the other side but that mortar is soft enough to dismantle that wall. And then the fact that I can feel positive about that and I feel hopeful about that and have some control over it. That feels good too. So, it's like a confidence booster. I haven't had confidence for too long.”

Jacky (40) | v15

“When these triggers and things happen, I think that that is the hardest thing for people. It takes time and the ability to trust that you can be with it, and it's not going to hurt you because the desire and the, immediate reaction to it is just, you know, carry on. And that's when that millisecond comes in between that stimulus and that immediate reaction, and I get that pause to be like, Oh, wait, no, I have a choice. And so, what I'm going to do here, that’s that magic moment. When it's the difference between I'm going to do what I've always done, or: I'm going to do something different this time. And I think that every time that that happens, it makes that thing a little bit different. That initial trigger loses some of its gumption or wiring, I’m literally rewiring the pathways there.”

Nick (41) | v15

“I hold value in holding space. Not just for my family but also for society and for myself. I'm worth it. I guess that's part of loving myself a little bit more and believe in myself more so I can handle that kindness. When you have parts of you that are taken away, things start to happen outside of your control. You start to lose a little bit of confidence to be able to control the rest of it and some of these involuntary things that started in July 2004. And then slowly, over time, reactions are too loud and not getting over that and not being able to control your sleep and not being able to, you know cope and deal with your problems in a normal way. Is it it's like a snowball effect you start to lose some value or some changes and you just kind of give up.”

*Theme 4 Relaxation, Calmness, Peace*

Rose (23y) | v9

“I can feel it. That's why it's so hard for me to explain. I can't tell you what's different about me and I can't give you an example. It's just like this really strong feeling of calmness. That's huge. And the part where I’m being kinder to myself. That's really good too. I think that's the biggest one. It's being calm.”

Jacky (40) | v15

“I made the analogy that I felt like my brain was running like, a dozen apps all the same time. I felt like that all the time. My boyfriend would tell me: you're exhausted all the time. And it was because my brain wouldn't stop. It would not stop this stuff. And I would say, going through this therapy, those are the subtle changes that aren't necessarily something like: Eureka, I wake up one morning and I’m changed. But those are the subtle changes that happen because over this process I have slept more than I probably slept in years. My body is legitimately just been like, No, you need to go to sleep. And I've just gone to sleep. And do I still have sleep disturbances? Yes, but they have gone down somewhat. But it’s almost like my body in some ways is trying to reset some of those things. My brain is trying to reset some of those things. Because those, those voices that were just loud, and I don't mean like super phrenic real voices, but you know what I mean? Those thoughts, that thought pattern, they slowly started to quiet down. They're not necessarily driving the car anymore. They're not even in the driver's seat. They've got their whole setup, they're comfortable, their coffees here, everything there and now you're driving the car for a while. Like, why don't you sit in the back. You don't have to leave. You can you can stay on the ride. Because you’re helpful. Sometimes, so stay, but, you're not going to drive the car anymore. You've driven the car for a really long time.”

Isa (43) | v15

“It's like having a clean house where everything's gone from the house. You know what I mean? I don't really want to move a whole bunch of furniture back in. It feels good, you know. So, I'm just leaving my mother outside and everybody, because I really like this. Quiet. You know, that's the other thing. My mind never stopped ever, and now it's just not going. I feel more relaxed.”

**Primary category 4 Interpersonal**

*Theme 1 Comfort*

Rose (23y) | v15

“I was stressing out, and he was trying to give me advice. And never before could I confidently say that somebody could give me advice, and I would sit there and try to listen to what they're saying. Usually I'm just like, no, you're wrong when you don't understand, but like I was actively, believing him and wanting his advice and I was able to see it from that perspective. You know, but like before I was so stuck that I couldn't listen to what they were saying.”

Nick (41) | v15

“Therapist: That's kind of the critical piece of your ability to love other people. And how it felt pretty clear yesterday for you, that that has such a big impact on the way that you connect with other people. So that the idea that your relationship with yourself has a direct impact on relationships with other people.”

Nick: “It's like a one-way glass. I can see how my relationship with myself impacts the quality of the relationship with others. But I never looked at it from the other way. The quality of my relationship with others can positively impact my relationship with myself.”

*Theme 2 Gratitude, Compassion, Empathy*

Nick (41y) | v15

“It kind of shed light on the idea of helping others. Like, let's put some light on dark spaces. That seems pretty cool. And so, I thought as I was lying in bed last night about at some point, like maybe after the study I would like to see if there's a way to volunteer, to sign up, or maybe look at the bar association and see if there's some way to do pro bono cases that involve doing something related to this kind to help others.”

Jacky (40) | v15

“That change in reaction of really trying to understand where other people are coming from. Like with my friend, that's one of my coworkers that has had that trauma and reacts pretty poorly at work a lot of time. It's like, I can see where a lot of his habits and reactions are coming from. And so, I can empathize more with him and I can handle it more. I can handle it differently, you know, in a more effective way. It's almost like managing that ego. It’s easier for me to handle his ego than most other people. It's almost like knowing the secret code. (…) What this drug does is it allows you to see things sometimes even from the other person's perspective. Not that you can 100% be in their mind, but also to understand maybe their reactions for things the way that they have come to things, and to really internalize it and be like Jesus Christ, man, how terrifying for you or how wonderful for you or whatever. And to have that for somebody else's, Oh, so beautiful. There's no better intimacy than that. You know what I mean? Like, there's no better intimacy than being able to say, f*ck, I see this from your perspective, and I really see it, I don't just like, Oh, I get it, but like, no, I totally get it, you know.”

Rico | v15

“I'm really Glad that Molly and I are together. I do feel like I'm in a space where I can start supporting her in a way that wasn't exactly possible before. We talked and the way that I would interact when we talked about like her day or our day together, I would say things like, Oh, well, you know, how was your day and I would just kind of go through the motions I notice. And that was, I'm glad that I'm not like, in that position anymore.

Therapist: “How do you do it now?”

Rico: “Now, I feel like I'm actually there and I am feeling that kind of empathy because before I knew that at this point you need to empathize and this is the time where you ask a question and it was just very check-the-box. But now, I am a good partner and what do what you should be doing. Which isn't to say that it was coming from a bad place. I wanted to help her but I just couldn't connect. It's exciting. I just feel like I’m starting to show up for people in a way that's so different and making friends in a way that I didn't think was possible, because I didn't think I was deserving of it.”

**Primary category 5 Connection**

*Theme 1 Union, Wider perspective*

Sophie (25y) | v9

“And then I took my trip to Georgia, in which I made the realization that I'm billions of years old, which is an awesome realization. Like, every once in a while, I'll like stop in the middle of what I'm doing at work and be like, remember you’re billions of years old? Which has been a super awesome kind of like, I feel like it grounds me like, hey, there's so much more in the universe than just what this one problem is right here. (...) There's a life source or an energy or a god. That's just continual love and respect for what is.”

*Theme 2 Inner healing intelligence*

Sophie (25y) | v15

“When you say, ‘the inner healer’ I think that the medicine (MDMA) allows something within you to find that awaiting ability to express it, whatever that something is. So, there's definitely an almost esoteric sense to it, for lack of a better term, there's some kind of magic to that, of you getting in that spot and be like, “hey, remember this?” And it's not always a good thing. But it's there. It's being brought up for a reason. And that to me is like, whoa. Yeah, truly It really is. It's like a moment of like, Oh, yeah, there's this memory. That's there for a reason. What is it bringing me? What is it teaching me and why is it still prevalent? What does it mean?”

Nick (41) | v13

“I think the interesting thing is like, for me, “the healer” isn't at all omniscient or kind of all-knowing or anything like that. I just have a little more perspective or a little more access.”

Therapist: “I think it was interesting also what you were saying about the healer yesterday. That the healer wasn't just the sergeant. It wasn't just the child view. It was like all the parts of you. Like almost like the whole healer. It is like this holistic version of all your different parts.”

Harry (50) | v15

“I was just lucky to be able to do that. I never thought before that I could have that like sort of intelligence about myself and my body or the ability to go through them and actually believe it. Like it's a fact. Those are things that I just couldn't even imagine. If you told me that I would feel that way after the study, I had never known what that meant.”

*Theme 3 Accessibility to emotions, in contact with feelings*

Rose (23y) | v15

“I feel like I've been feeling a lot more. Like, just a lot more feelings in general. The numbing is going away and the other day, I felt so sad. I felt so sad. Like, my heart was hurting. I was laying there. I had no clue why I felt so sad. And then I started thinking and I was like, you know, I didn’t felt this in so long. This is so good that I’m feeling this. I can just tell it's good. I feel like I'm getting a little bit closer to my feelings. Because feelings are very real. Before I was just, relying on thoughts, and now I feel connected to my feelings. So, I can say yeah, I'm sure because I'm feeling it like wow, really magic.”

Jacky (40) | v5

“Talking about those things that are difficult for me to talk about. And then also, they're only difficult for me to talk about them when I feel them. And that's what was so different because I wasn't just telling the story that I've told a million times. Because I can do that. Right now. I can separate it so well, but I can be like, I'm going to tell you the story in detail. And I'm not going to cry. I'm not going to get upset, because I legitimately can just block off all the feeling to it. But yesterday, that was impossible. Right? Like, even the times when I was talking and I was like, I'm going to keep it together. Like I literally couldn't, I was still feeling those emotions. Which is incredible. Because it's like, I finally outsmarted me. Great, because you know, that one part that has been doing what it's doing so long for survival has just been so conditioned to do that. The rest of the parts are just the weaker muscles, they’re just like, Oh, we could, but this one is the strongest. (…) The medicine (MDMA) for me, I felt more of an openness just in general to go to those places and certain memories and certain feelings that are attached to those memories, and allowing myself to feel those in a much more genuine way. If that makes sense. Yeah, whereas before, it's like I could think about things and even discuss them, but I was not allowing myself to feel them. It's like a total separation”

*Theme 4 Mind-body connection*

Sophie (25y) | v15

“So today driving home, I felt so much anxiety and fear and it intensified the closer I got to my apartment. It was resided in my chest and like the beating of my heart made me feel like I was going to suffocate. My shoulders were heavy, and I felt like my body would collapse under the strain of the anxiety and like my body would actually shut down and like would feel very felt really warm and you know, like that intensified heartbeat, and just like, a sense of panic of like, how am I? What do I even do in this situation? And it was crazy driving home feeling all this and then recognizing that feeling and be like, oh my gosh, like, this is just how I thought going home felt like for so long in my life and not even, you know, not being aware.”

Jacky (40) | v15

“You know, there's such a feeling separation between my mind and my body that I didn't really know I had. I truly didn't. Like I was dumb to that. Before going into this. If somebody would have been like, oh, the problem is, you're not connected here. I have a yoga teacher training certificate, right? If somebody told me that, I would have smiled to him and be like, oh, you're probably right and in my brain, I would think he’s a moron. But the truth is, that there was such a disconnect between those things. There became also this disconnect between me and everything around me. If that makes sense. That disconnect between my mind and body also became a disconnect with me and in my environment, my world. It was almost the same way. I thought that I was connecting with all these things. I thought I was enjoying being with Wilder, I thought I was in love with Andy. I thought I could do whatever X, Y or Z, but I wasn't feeling it so it made me feel very lonely. Like incredibly alone all the time. My number one feeling is just complete loneliness. That's what I've kind of felt, pain. It's just this pain of loneliness. I can tell you going into see my therapist like many times I'm just like, I feel alone. I feel untethered, I feel like I'm not connected to anyone. I feel like I am alone in the world. I feel even surrounded by people who are showing me so much love I feel objectively alone. And now more connected to yourself changes everything. So, all those loving people are still there, but we're more able to take it in.”

Rose (23) | v15

“I didn't feel very attached to my body anymore. And I kind of payed attention to that more. Because I feel like it was so hard for me even when you were asking me to pay attention to it; I hardly felt Anything. So now I can. I feel my body and my brain feels attached to my body. Wow. Yeah. So I can actually enjoy things now.”
